# Ganglioside-monosialic acid (GM1) prevents oxaliplatin-induced peripheral neurotoxicity in patients with gastrointestinal tumors

**DOI:** 10.1186/1477-7819-11-19

**Published:** 2013-01-25

**Authors:** Yanyun Zhu, Junlan Yang, Shunchang Jiao, Tiefeng Ji

**Affiliations:** 1 Department of Medical Oncology, Chinese PLA General Hospital, 28 Fuxing Road, Haidian District, Beijing, 100853, People’s Republic of China

**Keywords:** Gastrointestinal tumors, Ganglioside-monosialic acid, Oxaliplatin, Neurotoxicity

## Abstract

**Background:**

Oxaliplatin, an effective antineoplastic agent againstgastrointestinal tumors, can cause severe peripheral neurotoxicity, which seriously limits its clinical application. To date, there are no effective treatments for this complication. Ganglioside-monosialic acid (GM1) has been shown to protect neurons against injuries and degeneration. The aim of this study was to evaluate the effects of GM1 on preventing oxaliplatin-induced neurotoxicity in patients with gastrointestinal tumors.

**Methods:**

In this study, 120 patients with gastrointestinal tumors were enrolled, andthey received the treatment of XELOX (oxaliplatin and capecitabine) and FOLFOX4 (oxaliplatin, leukovolin and 5-fluorouracil). The patients were randomly divided into two groups, the experimental group and control group, with60 patients ineach. On the day chemotherapy was initiated, the experimental group received GM1 intravenously (100 mg once daily) for 3 days, while no neuroprotective agents were applied in the control group. The incidence rates and classification of neurotoxicity in the two groups were evaluated and the differences between the two groups were examined. Furthermore, whether GM1 affected the therapeutic effects of chemotherapy was also examined.

**Results:**

The grade of neurotoxicity in the experimental group was significantly lower than in the control group (*P*<0.05, Mann–Whitney U test). The probability of occurrence of low-grade neurotoxicity (grade 0 and 1) in the experimental group was higher than that in the control group (logistic ordinal regression); whereas the probability of occurrence of high-grade neurotoxicity (grade 2 and 3) in the experimental group was lower than in the control group (logistic ordinal regression).

**Conclusion:**

The data suggested that GM1 could reduce the grade of oxaliplatin-induced neurotoxicity and was an effective neuroprotective agent against oxaliplatin-induced high-grade neurotoxicity in patients with gastrointestinal tumors.

## Background

Oxaliplatin is one of the major antineoplastic agents in the treatment of gastrointestinal tumors [1]. Its major toxic side effect is peripheral neurotoxicity, which mainly causes sensory disturbances in the lower extremities [2]. Oxaliplatin-induced neurotoxicity has been classified into two types: acute and chronic neurotoxicity. The mechanism underlying oxaliplatin-induced acute neurotoxicity is that oxaliplatin affects the voltage-gated sodium channels in the surface of the cell membrane of nerve fibers [3]. In five clinical trials involving 210 patients, reversible signs and symptoms of acute neuropathy were found to occur in 82 to 98% of treated patients [4]. The mechanism underlying oxaliplatin-induced chronic neurotoxicity is that oxaliplatin inhibits the synthesis of rRNA in the nucleolus of neuronal cell bodies, resulting in morphological change and damage of sensory neurons [5]. Grothey and Cersosimo reportedthat the incidence of chronic neurotoxicity (grade 3/4) was about 16% [6,7].

At present, oxaliplatin-induced acute neurotoxicity is mainly prevented by regulating sodium channels inclinic. The approaches to regulate sodium channels include sodium channel blockers, and calcium and magnesium infusions. However, itremains controversial whether calcium and magnesium infusions can reduce the efficiency of oxaliplatin [8,9]. It is reported that glutathione can prevent accumulation of platinum agents in the dorsal root ganglion (DRG), and thus glutathione can be used to prevent and treat oxaliplatin-induced chronic neurotoxicity [10]. Furthermore, amifostine can also prevent neurotoxicity induced by chemotherapy drugs such as paclitaxel and cisplatin [11]. However, these drugs exhibit limited efficacy in the prevention and treatment of oxaliplatin-induced neurotoxicity. Therefore, there is an urgent need to develop new drugs to prevent and treat oxaliplatin-induced neurotoxicity [12].

Ganglioside-monosialic acid (GM1) is a type of glycosphingolipid with one sialic acid. GM1 is located on the outer layer of the plasma membrane, and plays a vital role in neurogenesis, nerve development, differentiation and repair after injury [13]. Currently, preclinical research has demonstrated the potential neuroprotective effects of GM1 in central nervous system diseases and Parkinson’s disease [14-16]. Notably, GM1 is also used in the treatment of peripheral neuropathy such as diabetic peripheral neuropathy in preclinical animal models, owing to its superior neuroprotective effects and function of nerve repair [17].

The mechanisms whereby GM1 exert their superior neuroprotective effects and function of nerve repair include: 1. GM1 can recover the activity of Na^+^-K^+^-ATP enzyme and Ca^2+^-Mg^2+^-ATP enzyme, and promote the regeneration and recovery of nerves [18]. 2. GM1 can interact with nerve growth factor (NGF) to promote the regeneration of nerves [19]. 3. GM1 can inhibit the lipid peroxidation and remove oxygen free radicals, reducing the damage of nerve cells [20]. 4. GM1 can affect the morphological changes of DRG neurons and protect the DRG neurons from damage induced by excitotoxic glutamate [21]. Considering DRG neurons are a target for neurotoxicity induced by oxaliplatin, GM1 may exert its neuroprotective effects by affecting DRG neurons. In recent years, GM1 has been used in the prevention of neurotoxicity induced by chemotherapy drugs such as paclitaxel in preclinical animal models [22]. However, there are no reports of the effects of GM1 in the prevention of oxaliplatin-induced peripheral neurotoxicity.

In this study, 120 patients with gastrointestinal tumors were enrolled and received the treatment of XELOX and FOLFOX4. The patientswere randomly divided into two groups, the experimental group and control group,with 60 patients in each. The incidence rates and classification of neurotoxicity in the two groups were evaluated. The aim of this study was to evaluate the effects of GM1 on preventing oxaliplatin-induced neurotoxicity in patients with gastrointestinal tumors.

## Methods

### Patients

In this study, 120 patients were enrolled with gastrointestinal tumors (gastric cancer or colorectal cancer). They received chemotherapy containing oxaliplatin at the Department of Medical Oncology, Chinese PLA General Hospital, during a 1-year period from December 2010 to December 2011. All the patients had an Eastern Cooperative Oncology Group (ECOG) performance status of 0 to 1. Forty-nine patients (40.8%) underwent adjuvant chemotherapy after surgery for gastric cancer. Seven patients (5.8%) underwent first-line chemotherapy for gastric cancer. Twenty-two patients (18.3%) underwent adjuvant chemotherapy after surgery for rectal cancer. Eleven patients (9.2%) underwent first-line chemotherapy for rectal cancer. Eighteen patients (15%) underwent adjuvant chemotherapy after surgery for colon cancer. Thirteen patients (10.8%) underwent first-line chemotherapy for colon cancer.

The patients were randomly divided into two groups, the experimental group and control group, with 60 patients in each. The assignation to the two groups was generated by using a computer program, Randomlogue, produced by the Department of Social Statistics, Southampton University, UK. Only the fourth author, TFJ, who applied the treatment, was aware of the group assignment of each patient, and TFJ did not participate in any of the subsequent evaluation phases. The first author, YYZ, and second author, JLY,undertook the evaluation, and YYZ and JLY were blinded to the group assignment of each patient.

The further criteria for inclusion in this study were: aged between 18 to 75 years and life expectancy >3 months; ECOG performance status of 0 to 1; tumors demonstrated as gastric cancer or colorectal cancer by pathology, and treated by chemotherapy containing oxaliplatin; routine blood tests performed 0 to 3 days before chemotherapy (absolute neutrophil ≥1.5 × 10^9^/L, platelets ≥100 × 10^9^/L and hemoglobin ≥9 g/dL); hepatic function test (aspartate aminotransaminase and alanine aminotransferase lower than 1.5 fold of the upper limit of normal values, and lower than 2.5 fold of the upper limit of normal values in patients with known hepatic metastases); renal function test (a calculated creatinine clearance rate <45 mL/min); and without any neurotoxicity (grade 0).

Patients with signs of malnourishment or >10% weight loss in the previous 6 weeks, or other serious concomitant disorders were excluded from the therapy. Patients were discontinued from the therapy in the case of evidence of progressive disease or unacceptable toxicity despite dose adjustment.

This study was conducted according to the International Conference on Harmonisation (ICH) Good Clinical Practice (GCP) guidelines, including obtaining written informed consent from all patients. This study was registered at the Department of Scientific Research, Chinese PLA General Hospital (register number: 2007–3048).

### Sample size

The sample size was calculated based on significant neurotoxicity relief. Considering a 0.05 two-sided significance level, a power of 80% and an allocation ratio of 1:1, 55 patients were required in each group [23]. Allowing for a 10% attrition/non-compliance rate, 60 subjects were required.

### Medication

All 120 patients underwent oxaliplatin-containing chemotherapy consisting of XELOX (oxaliplatin 130 mg/m^2^ Vdday 1; capecitabine 850 to 1000 mg/m^2^twice dailyday 1 to 14; repeat day 21) and FOLFOX4 (oxaliplatin 85 mg/m^2^ Vd day 1; leukovolin 200 mg/m^2^ Vdday 1 to 2; 5-fluorouracil 400 mg/m^2^intravenouslyday 1 to 2, 600 mg/m^2^by continuous intravenous infusionfor 22 hours day 1 to 2; repeat day 14). On the day chemotherapy was initiated, the experimental group received GM1 (Qilu Pharmaceutical Co., Ltd, Shandong, China) intravenously (100 mg once daily, according to the manufacture’s protocols), before chemotherapeutics administration for 3 days; while no neuroprotective agents were applied in the control group.

### Objectives

This study seeks to evaluate whether GM1 is an effective neuroprotective agent against oxaliplatin-induced high-grade neurotoxicity in patients with gastrointestinal tumors.

### Neurotoxicity grading scales

Fourteen (for the 2-week regimen) or twenty-one (for the 3-week regimen) days after each chemotherapeutic cycle, the incidence rates and classification of neurotoxicity in the two groups were comprehensively evaluated using the National Cancer Institute (NCI)-Sanofi criteria (Table 1) [24]. The neurotoxicity was recorded after each chemotherapeutic cycle and the most severe neurotoxicity in all chemotherapeutic cycles was recorded as the final recorded neurotoxicity of each patient. 

**Table 1:** Neurotoxicity grading scales

**Standard setting unit**	**Grade 1**	**Grade 2**	**Grade 3**	**Grade 4**
NCI-Sanofi criteria	Paresthesias or dysesthesias of short duration that resolve and do not interfere with function	Paresthesias or dysesthesias interfering with function but not activities of daily living	Paresthesias or dysesthesias with pain or functional impairment that also interfere with activities of daily living	Persistent paresthesias or dysesthesias that are disabling or life-threatening

NCI, National Cancer Institute.

### Response Evaluation Criteria In Solid Tumors (RECIST)

The enrolled patients were classified into two categories: adjuvant chemotherapy after surgery and first-line chemotherapy. The clinical evaluation of the patients undergoing first-line chemotherapy, who all had measurable target lesions, was performed according to the RECIST proposed by Therasse *et al.*[25], which described: Complete Response (CR): disappearance of all target lesions; Partial Response (PR): at least a 30% decrease in the sum of the longest diameter (LD) of target lesions, taking as reference the baseline sum LD; Stable Disease (SD): neither sufficient shrinkage to qualify for PR nor sufficient increase to qualify for PD, taking as reference the smallest sum LD since the treatment started; Progressive Disease (PD): at least a 20% increase in the sum of the LD of target lesions, taking as reference the smallest sum LD recorded since the treatment started or the appearance of one or more new lesions.

### Statistical analysis

All data in this study were processed using SPSS 13.0 software (IBM, Armonk, NY, USA). For group comparisons, unordered categorical variables were compared using chi-square (χ^2^) test or Fisher’s exact test, measurement data were compared using Student’s *t*-test, and ordinal variables of multi-classification were compared using Mann–Whitney U test. Logistic ordinal regression was used to determine correlations in classification of neurotoxicity between the experimental group and control group. A *P* value <0.05 was considered statistically significant.

## Results

The general and clinicopathological characteristics of the 120 patients are shown in Table 2. There were no significant differences in gender, diagnosis, therapeutic approaches, smoking or drinking status, complicated diseases and chemotherapy regimens between the two groups (Table 2). The age of patients ranged from 21 to 74 years, with an average age of 54.96 years. In the experimental group, the fewest and most chemotherapeutic cycles were 2 and 12, respectively, with an average cycle of 5.88. The cumulative oxaliplatin dose (130 mg/m^2^ (XELOX) or 85 mg/m^2^ (FOLFOX4) × chemotherapeutic cycles) of the experimental group ranged from 227.27 to 1103.90 mg/m^2^. In the control group, the fewest and most chemotherapeutic cycles were 3 and 12, respectively, with an average cycle of 6.63. The cumulative oxaliplatin dose of the control group ranged from 384.62 to 1077.84 mg/m^2^. There was no significant difference in average cumulative oxaliplatin dose between the experimental group and control group (692.08 ± 196.08 mg/m^2^vs. 740.83 ± 222.65 mg/m^2^, mean ± SD, n = 60, *P* = 0.206, Student’s *t*-test). There were no significant differences in age and chemotherapeutic cycles between the two groups (Table 3).

**Table 2:** General and clinicopathological characteristics of patients (n = 120)

**Groups**		**Experimental group**		**Control group**		***P *value**
		**Frequency**	**Percentage (%)**	**Frequency**	**Percentage (%)**	
Gender	Male	49	81.67	44	73.33	0.274^a^
	Female	11	18.33	16	26.67	
Diagnosis	Colon cancer metastases	6	10	6	10	0.066^a^
	Postoperative colon cancer	8	13.33	11	18.33	
	Gastric cancer metastases	4	6.67	3	5	
	Postoperative gastric cancer	32	53.33	17	28.33	
	Rectal cancer metastases	3	5	7	11.67	
	Postoperative rectal cancer	7	11.67	16	26.67	
First-line therapy	No	44	73.33	43	71.67	0.838^a^
	Yes	16	26.67	17	28.33	
Smoking	No	38	63.33	39	65.00	0.849^a^
	Yes	22	36.67	21	35.00	
Drinking	No	46	76.67	40	66.67	0.224^a^
	Yes	14	23.33	20	33.33	
Diabetes	No	51	85.00	54	90.00	0.408^a^
	Yes	9	15.00	6	10.00	
Chemotherapy regimen	FOLFOX4	13	21.67	20	33.33	0.152^a^
	XELOX	47	78.33	40	66.67	
Clinical evaluation	CR	1	1.67	1	1.67	nd^b^
	PD	2	3.33	3	5.00	
	PR	7	11.67	3	5.00	
	SD	6	10.00	10	16.67	
	Assistance	44	73.33	43	71.67	

^a^Comparisons performed using χ^2^ test; ^b^The sample number is small and comparisons are not done. CR, Complete Response;nd, not done; PD,Progressive Disease; PR, Partial Response;SD,Stable Disease.

**Table 3:** Comparison of age and chemotherapeutic cycles between the two groups

	**Experimental group**	**Control group**	***P *value**^a^
Age	55.10	54.83	0.894
Chemotherapeutic cycles	5.88	6.63	0.055

^a^Comparisonsperformed using Student’s *t*-test.

### Neurotoxicity evaluation

After chemotherapy, the incidence rates and classification of neurotoxicity in the two groups were comprehensively evaluated using the neurotoxicity grading scales, and the differences between the two groups were examined. All patients exhibited grade 0 to 3 neurotoxicity, and no patients exhibited grade 4 neurotoxicity. In our study, the incidence of neurotoxicity (grade 1, 2 and 3) in the experimental group was 68.33%; while that in the control group was 78.33%. The incidence rates of grade 0 neurotoxicity in the experimental and control group were 31.67% and 21.67%, respectively. Grade 1 was 33.33% and 26.67%, respectively. Grade 2 was 26.67% and 23.33%, respectively. Grade 3 was 8.33% and 26.33%, respectively. The grade of neurotoxicity in the experimental group was significantly lower than in the control group (*P* = 0.021, Mann–Whitney U test) (Table 4). Notably, the probability of occurrence of high-grade (grade 2 and 3) neurotoxicity in the experimental group was lower than that in the control group (grade 2: 21.64% vs. 29%, grade 3: 12.95% vs. 24.32%, logistic ordinal regression). Owing to the lower probability of occurrence of high-grade (grade 2 and 3) neurotoxicity in the experimental group than the control group, the probability of occurrence of low-grade (grade 0 and 1) neurotoxicity in the experimental group was higher than that in the control group (grade 0: 33.77% vs. 19.09, grade 1: 31.64% vs. 27.58%, logistic ordinal regression) (Tables 5 and 6). There were significant differences in the probability of occurrence of neurotoxicity between the two groups (*P* = 0.041, χ^2^ test) and the probability of occurrence of grade 3 neurotoxicity was significantly lower in the experimental group than in the control group (*P* = 0.005, χ^2^ test).

**Table 4:** Comparison of the grade of neurotoxicity between the two groups

**Grade of neurotoxicity**	**Experimental group**		**Control group**		***P *value**^a^
	**Frequency**	**Percentage (%)**	**Frequency**	**Percentage (%)**	
0	19	31.67	13	21.67	0.021
1	20	33.33	16	26.67	
2	16	26.67	14	23.33	
3	5	8.33	17	28.33	

^a^Comparisons performed using Mann–Whitney U test.

**Table 5:** **Analysis of the relationship between the two groups in the grade of neurotoxicity using logistic ordinal regression**^**a**^

		**Regression coefficient**	**Standard error**	**Wald**	**Degree of freedom**	***P *value**	**95% confidence interval**	
							**Lower limit**	**Upper limit**
	[Grade = 0.00]	−1.444	0.281	26.388	1	0.000	−1.995	−0.893
Dependent variable	[Grade =1.00]	−0.133	0.246	0.292	1	0.589	−0.616	0.350
	[Grade = 2.00]	1.135	0.274	17.124	1	0.000	0.598	1.673
Independent variable	[Class = 1.00]	−0.770	0.334	5.317	1	0.021	−1.425	−0.116
	[Class = 2.00]	0.000						

^a^The logistic model was successfully constructed, demonstrated by χ^2^= 5.402 and *P* = 0.020. The regression coefficient was −0.770, thus the odds ratio of neurotoxicity in the experimental group was 2.16, suggesting the probability of occurrence of low-grade neurotoxicity (grade 0 and 1) in the experimental group was significantly higher than that in the control group.

**Table 6:** **Predicative probability of neurotoxicity in the two groups**^**a**^

**Groups**		**Grade of neurotoxicity**			
		**0**	**1**	**2**	**3**
	Observed value	19	20	16	5
Experimental group	Predicative value	20.26	18.98	12.98	7.77
	Predicative probability	33.77	31.64	21.64	12.95
	Observed value	13	16	14	17
Control group	Predicative value	11.46	16.55	17.40	14.59
	Predicative probability	19.09	27.58	29.00	24.32

^a^The probability of low-grade (grade 0 and 1) neurotoxicity occurring in the experimental group was higher than in the control group, while the probability of high-grade (grade 2 and 3) neurotoxicity occurring in the experimental group was lower than in the control group.

In the experimental group, six patients exhibited grade 2 to 3 neurotoxicity 1 to 3 months after chemotherapy; but only one patient exhibited such neurotoxicity in the control group. During the treatment, four cases of severe allergy occurred, with three in the experimental group and one in the control group. The severe allergy all occurred during the administration of oxaliplatin and after its repeated administration, with associated symptoms of breathing difficulties, sweating, pale and cyanotic lips, which were relieved within 20 minutesfollowing oxygen and anti-allergy treatment. Two patients underwent surgical treatment after chemotherapy and exhibited severe neurotoxic symptoms after surgery. One patient exhibited torpid reaction and memory loss. Twenty-five percent of patients exhibited decreased visual acuity and/or hypogeusia. There was no significant difference in the incidence rates of decreased visual acuity and/or hypogeusia between the two groups (Table 7). The median duration of remission of decreased visual acuity and/or hypogeusia was still in follow-up.

**Table 7:** Comparison of the incidence rates of decreased visual acuity and/or hypogeusia between the two groups

**Symptoms**	**Experimental group**		**Control group**	**Sum**	***P *value**^a^
Yes	Frequency	44	46	90	0.673
	Percentage (%)	73.33	76.67	75.00	
No	Frequency	16	14	30	
	Percentage (%)	26.67	23.33	25.00	
Sum	Frequency	60	60	120	
	Percentage (%)	100.00	100.00	100.00	

^a^Comparisons performed using χ^2^ test.

## Discussion

Oxaliplatin-induced neurotoxicity is a common, potentially severe and dose-limiting adverse effect of cancer treatment [26]. The characteristics of oxaliplatin-induced neurotoxicity are related to dose intensity and cumulative dose. Neurotoxicity can profoundly affect the qualityoflife, often compelling clinicians to lower the chemotherapy regimen, consequently limiting therapeutic efficacy [27]. Oxaliplatin-induced neuropathy is of two types: acute and chronic. Acute neuropathy was believed to reflect a state of peripheral nerve hyperexcitability that likely represents a transient oxaliplatin-induced impairment of ion channels, while the chronic treatment induces an axonal neuropathy similar to the other platinum-based drugs [28]. Strategies to ameliorate oxaliplatin neurotoxicity include the use of several ‘neuroprotective’ drugs, such as reduced glutathione [29], amifostine [30], andcalcium and magnesium infusion. [31]. GM1 is used in the treatment of peripheral neuropathy such as diabetic peripheral neuropathy in preclinical animal models, owingto its superior neuroprotective effects and function of nerve repair. To our knowledge, the clinical use of GM1 in the prevention of oxaliplatin-induced neurotoxicity has not yet been investigated.

Four of the enrolled patients (3.33%) presented with dyspnea, which was a little higher than that of a previous study (1 to 2%) [32]. The dyspnea of these four patients was relieved by symptomatic treatment and they underwent complete chemotherapy after intravenous drip was slowed. Only one patient developed dyspnea again when undergoing chemotherapy and the other three patients did not develop symptoms of dyspnea again. It is reported that multi-infusion and prolongation of the infusion time could reduce the neurotoxicity of oxaliplatin [33,34]. The dyspnea that occurred in the patients may be associated with the rapid infusion of oxaliplatin. Whether GM1 has causative or therapeutic effects on dyspnea is not clearand the effects of GM1 on dyspnea induced by oxaliplatin should be observed in a clinical trial of a sufficiently larger sample size.

Two patients who underwent surgery after chemotherapy presented with postoperative aggravation of neurotoxicity, which was consistent with previous reports that surgery could aggravate neurotoxicity induced by oxaliplatin [35]. In the control group, one patient receiving the cumulative dose of oxaliplatin of 1800 mg (1065.09 mg/m^2^) presented with torpid reaction, loss of memory and grade 3 neurotoxicity. The results obtained by Lehky showed that rare neurotoxicity such as urine retention, Lhermitte’s sign and reversible posterior leukoencephalopathy syndrome could occur when the cumulative dose of oxaliplatin exceeded 1000 mg per patient [28]. Twenty-five percent of patients exhibited decreased visual acuity and/or hypogeusia. There was no significant difference in the incidence rates of decreased visual acuity and/or hypogeusia between the two groups, suggesting that GM1 could not prevent the toxic effects of oxaliplatin on vision and taste receptors.

In our study, the total incidence of neurotoxicity in the experimental group was 68.33%, while that in the control group was 78.33%. However, the grade of neurotoxicity in the experimental group was significantly lower than in the control group (*P* = 0.021). Notably, the probability of occurrence of high-grade (grade 2 and 3) neurotoxicity in the experimental group was lower than in the control group (Table 6). There were significant differences in the probability of occurrence of neurotoxicity between the two groups (*P* = 0.041, χ^2^ test) and the probability of occurrence of grade 3 neurotoxicity was significantly lower in the experimental group than in the control group (*P* = 0.005, χ^2^ test). In the experimental group, six patients did not present with neurotoxicity during treatment. However, they presented with grade 2 or 3 neurotoxicity 1 to 3 months after chemotherapy. We speculated that this phenomenon was due to the short-term application of GM1. Owing to limited length of stay, the usage time of GM1 per patient was only 3 days in this study, leading to only partially preventive effects of GM1 exerted on patients.

In this study, the drugs which were used to prevent oxaliplatin-induced neurotoxicity mostly exhibited limited efficacy. Notably, calcium and magnesium infusion was reported to effectively decrease the incidence of chronic, cumulative, grade 2 or greater oxaliplatin-induced neurotoxicity in a non-randomized and retrospective study (*P* = 0.038). However, no effect on acute, cold-induced neurotoxicity was found. No substantial differences in adverse effects were noted between calcium/magnesium and placebo. Comparing the results from these trials may be difficult or impossible because of the use of different dosages and regimens of treatment, and the lack of standardization of the methods used in evaluating the extent or the incidence of neurotoxicity.

## Conclusion

In summary, these data suggest that GM1 could reduce the grade of oxaliplatin-induced neurotoxicity and was an effective neuroprotective agent against oxaliplatin-induced high-grade neurotoxicity in patients with gastrointestinal tumors. This study points to the potential of GM1 in reducing the oxaliplatin-induced high-grade neurotoxicity in patients with gastrointestinal tumors.

## Abbreviations

CR: Complete Response; DRG: Dorsal root ganglion; ECOG: Eastern Cooperative Oncology Group; GM1: Ganglioside-monosialic acid; LD: Longest diameter; NCI: National Cancer Institute; NGF: Nerve growth factor; PD: Progressive Disease; PR: Partial Response; SD: Stable Disease; Vd: Volume of distribution.

## Competing interests

The authors declare that they have no competing interests.

## Authors’ contributions

All authors have contributed substantially to the study. YYZ contributed to the design of the study, recruitment of patients, analysis of data and writing of manuscript. SCJ contributed to the conception and design of the study. JLY and TFJ gave contributions in the recruitment of patients. All authors read and approved the final manuscript.
